# A Qualitative Assessment of Community Acceptability and Its Determinants in the Implementation of Minimally Invasive Tissue Sampling in Children in Quelimane City, Mozambique

**DOI:** 10.4269/ajtmh.22-0343

**Published:** 2023-04-10

**Authors:** Amilcar Magaço, Yara Alonso, Maria Maixenchs, Contardo Ambrosio, Antonio Sitoe, Pio Vitorino, Dianna Blau, Mischka Garel, Robert Breiman, Agbessi Amouzou, Quique Bassat, Inacio Mandomando, John Blevins, Khatia Munguambe

**Affiliations:** 1 Centro de Investigação em Saúde de Manhiça, Maputo, Mozambique;; 2 ISGlobal, Hospital Clínic - Universitat de Barcelona, Barcelona, Spain;; 3 Emory Global Health Institute, Atlanta, Georgia;; 4 Johns Hopkins Bloomberg School of Public Health, Baltimore, Maryland;; 5 ICREA, Barcelona, Spain;; 6 Pediatrics Department, Hospital Sant Joan de Déu, Universitat de Barcelona, Esplugues, Barcelona, Spain;; 7 Consorcio de Investigación Biomédica en Red de Epidemiología y Salud Pública, Madrid, Spain;; 8 Instituto Nacional de Saúde, Ministério da Saúde, Maputo, Mozambique;; 9 Faculdade de Medicina, Universidade Eduardo Mondlane, Maputo, Mozambique

## Abstract

The Countrywide Mortality Surveillance for Action project aims to implement a child mortality surveillance program through strengthening vital registration event reporting (pregnancy, birth, and death) and investigating causes of death (CODs) based on verbal autopsies. In Quelimane (central Mozambique), Minimally Invasive Tissue Sampling (MITS) procedures were added to fine-tune the COD approaches. Before the implementation of MITS, an evaluation of the acceptability and ethical considerations of child mortality surveillance was considered fundamental. A socio-anthropological study was conducted in Quelimane, using observations, informal conversations, semi-structured interviews, and focus group discussions with healthcare providers, nharrubes (traditional authorities who handle bodies before the funeral), community and religious leaders, and traditional birth attendants to understand the locally relevant potential facilitators and barriers to the acceptability of MITS. Audio materials were transcribed, systematically coded, and analyzed using NVIVO12^®^. The desire to know the COD, intention to discharge the elders from accusations of witchcraft, involvement of leaders in disseminating project information, and provision of transport for bodies back to the community constitute potential facilitators for the acceptability of MITS implementation. In contrast, poor community mobilization, disagreement with Islamic religious practices, and local traditional beliefs were identified as potential barriers. MITS was considered a positive innovation to determine the COD, although community members remain skeptical about the procedure due to tensions with religion and tradition. Therefore, the implementation of MITS in Quelimane should prioritize the involvement of a variety of influential community and religious leaders.

## INTRODUCTION

Despite reductions over the past two decades, child mortality remains unacceptably high, particularly in low-income settings in sub-Saharan Africa, where 1 in 13 children dies before age 5, a rate that is 16 times higher than the average ratio of 1 in 199 in high-income countries.^1^ In Mozambique, the child mortality rate fell gradually from 190.8 deaths per 1,000 live births in 1998 to 73.2 deaths per 1,000 live births in 2018.^1^

An accurate understanding of global child mortality and health is severely limited by inadequate methods and measurements. Less than 20% of the 192 countries in the world have high-quality death registry data, and more than one-third do not have any specific mortality registry data.^2^ For this reason, tracking the mortality of under-five children is at the forefront of public health priorities. In low- and middle-income countries (LMICs), including Mozambique, children often die without a documented medical history and are often buried before the cause of death (COD) determination has been conducted.^3^^,^^4^

The Countrywide Mortality Surveillance for Action (COMSA) project is an initiative that aims to determine the causes of under-five mortality, including stillbirths, in Mozambique by measuring and monitoring mortality and COD utilizing verbal autopsies, a methodology widely used in areas where death certification remains inaccurate and with some limitations.^5^^,^^6^ In Zambézia province (central Mozambique), the surveillance activities include the additional implementation of minimally invasive tissue sampling (MITS), which is considered a valuable tool for COD investigation and for generating data to prioritize research and prevention strategies aimed at reducing child deaths in under-resourced settings.^4^^,^^7^^,^^8^ The MITS consists of a series of post-mortem punctures using fine biopsy needles aiming to obtain tissue samples and body fluids from a corpse within the first hours after death, which are then submitted for a thorough histopathological and microbiological investigation of the underlying COD.^4^^,^^7^

This technique is being used in several contexts in LMICs and appears to be promising in terms of its greater acceptability because it is considered a fast, simple, and more user-friendly technique that can be performed by minimally trained staff, although there are factors considered to be important barriers that may put its acceptability at risk.^9^^–^^11^ Existing evidence on the anticipated and experienced acceptability of the MITS procedure thus demonstrates the importance of recognizing the cultural mores and practices of the context where MITS is implemented.^9^^,^^12^

Death and related post-mortem procedures are embedded in complex social, cultural, and religious environments.^9^^,^^10^ In the case of Zambézia province (central Mozambique), the existence of myths, rumors, and negative perceptions of certain public health initiatives, as was the case with cholera control,^13^ could challenge the success of the implementation of MITS in this setting. Acknowledging this risk, it is critical to understand local attitudes and perceptions in relation to death as well as the potential barriers and facilitators to the uptake of interventions involving post-mortem procedures, which could determine the acceptability of the implementation of MITS in Quelimane.

## MATERIALS AND METHODS

### Study design.

This was a qualitative study that was conducted as part of the formative research to inform the COMSA program for investigating COD in Mozambique and other LMICs. COMSA is a sample registration system with community surveillance assistants who prospectively report birth, death, and COD data from a representative sample of communities in the country.^14^ Following an ethnographic approach, the study sought to understand how people in this region form and sustain their experiences and customs in relation to death. To do so, we explored this phenomenon from the socio-cultural perspective to understand the meaning and local practices related to health, disease, and death, in the view of later on incorporating a phenomenological approach to capture individuals’ understandings of the meanings of death of children embedded in their own lived experiences.^15^^,^^16^

### Study site and population.

**Figure 1.**
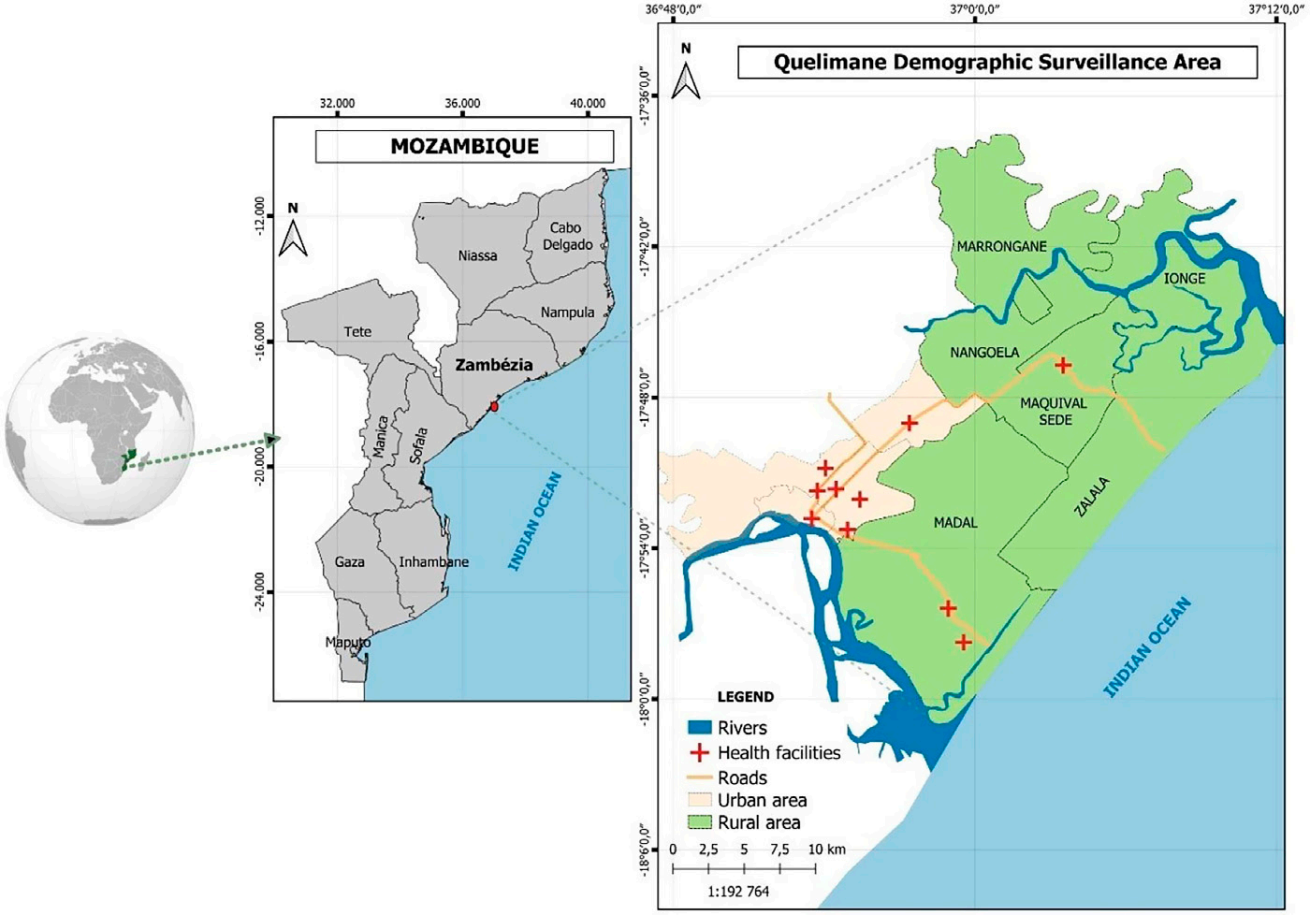
Map of study sites (Quelimane).

This study was conducted in Quelimane city, the capital of Zambézia province, central Mozambique. Quelimane is a district covering an area of 117 km^2^, with 193,343 inhabitants. It is located by the Bons Sinais River and about 20 km from the Indian Ocean (Figure 1). The main economic activities are fishing and agriculture. The population is mostly of Chuabo ethnicity, the dominant ethnic group, and Christianity is the main religion (60.2%), although a considerable part of the population (18.9%) is Muslim.^17^ The district, which administers the powers of the central government, incorporates a municipality with an elected local government with five Urban Administrative Posts. These administrative posts are designated in numbers, and most of them are rural areas where the predominant occupations are artisanal fishing and agriculture. Quelimane hosts 21 health facilities, including 17 health centers, two peripheral health posts, a general hospital, and a central hospital (tertiary level). The Central Hospital of Quelimane (HCQ) was also included as the point for selection of some key informants.

### Target groups and sampling.

This study targeted individuals with relevant roles and experiences related to the study questions (i.e., direct experience with caring for dead children’s bodies or with events related to the death of children and/or stillbirths). Thus, study participants consisted of community leaders (a community representative with central or local government legitimacy and/or influence and power over communities). Such power can be political, either by election or appointment (e.g., neighborhood secretaries, heads of blocks) or traditional by lineage (e.g., regulators); traditional birth attendants (who care for pregnant women, support them when they deliver outside health facilities, and care for newborns); nharrubes (traditional authorities that are in charge of washing the bodies and lead prayers before the funeral); and health care providers, such as medical doctors (pediatrician, neonatologist, and obstetrician); nurses and hospital support teams were included as well. These target groups were purposively selected to include diverse perspectives on the experience of caring for dead children’s bodies. During data collection we used snowball sampling to identify participants from each group. Snowball sampling was continued until the study team concluded that saturation had been reached and there was no need to interview more participants.

### Data collection.

We conducted a combination of qualitative data collection strategies. To better understand the local context, we carried out transect walks around the community accompanied by observations and informal conversations while identifying potential participants. During these observations and informal conversations, social science assistants generated field notes on participants’ behavior and reactions when discussing issues related to child mortality in the community. Focus group discussions (FGDs) were carried out with community leaders and traditional birth attendants (TBAs), and semi-structured interviews (SSIs) were conducted with nharrubes and health professionals directly linked to the HCQ in the pediatric, obstetrics, and maternity services and with nharrubes. Data were collected by four social science assistants, specifically trained for the purposes of the study, and one social science researcher, who also acted as the overall study coordinator. The study was overseen by a senior social scientist based remotely. Each data collection strategy followed its own topic guide or script, mostly formed by open-ended questions about the death of children and procedures for the care of a deceased child’s body. These general questions served to unravel potential alignments and tensions between current local practices and the procedures proposed by the mortality surveillance. Specifically, they sought to identify the perceived relevance and interest in knowing the COD, perceptions about the objectives of the MITS, and the role of community leaders and heads of families in caring for the bodies of deceased children. The SSIs focused on individual perspectives based on own experiences, whereas the FGDs explored the perceptions and experiences of the community at large, also taking into account local norms and values. The FGDs were composed of a minimum of 8 and a maximum of 12 participants belonging to the same target group.

Data collection was carried out in Portuguese or in the local language (Chuabo), depending on participants’ preference. Semi-structured interviews and FGDs lasted approximately 60–90 minutes, and informal conversations lasted approximately 20–30 minutes and took place during walks around the communities while identifying potential study participants.

### Data analysis.

Semi-structured interviews and FGDs were digitally recorded and fully transcribed by three experienced transcribers who were fluent in Chuabo and received specialized training in transcription techniques based on the study’s standards. Interviews conducted in Chuabo were simultaneously translated into Portuguese during transcription. The notes from informal conversations and observations were digitized by social science assistants after their return from fieldwork and were later triangulated with the SSI and FGD data during the analysis.

Data analysis was performed in two stages. The first stage was the pre-analysis of the field notes, which led to the development of analytic categories, while the interviews and focus group discussions were still being transcribed verbatim and to the to the definition of the theoretical saturation of data. The second stage was the coding and analysis of interview transcripts. In this phase, new categories were added, removed, and changed. This process was done using NVIVO12^®^ software (QSR International, Inc., Melbourne, Australia), which supported not only coding but also extraction of coded text, organization of categories and subcategories in a codebook, and establishment of relationships between ideas. Codifications were subjected to content analysis summarized and tabulated into a matrix format using MS Excel for framework and content analysis in a word sheet. The coding of the transcripts on NVivo was conducted independently by two coders hired for the purpose and a junior investigator. The two sets of codes were compared to ensure consistency.

### Ethical aspects.

The study followed a protocol that received ethical approval (CIBS-CISM/013/2018) by the Institutional Committee on Bioethics in Health of the Manhiça Health Research Center (CIBS-CISM). Administrative authorizations were provided by the Ministry of Health, the Zambézia Provincial Health Directorate, and the Municipal Authority of Quelimane City. Authorization to engage with community members and perform the study was also first requested from neighborhood secretaries and the heads of each administrative post included in the study.

## RESULTS

### Socio-demographic data.

In total, 113 people participated in the study. Twenty-six SSIs were conducted with 16 healthcare providers and 10 nharrubes, and 11 FGDs were organized involving 43 community leaders and 44 TBAs from the different administrative posts included in the study. Table 1 shows the distribution of the participants per target group and data collection tool.

**Table 1:** Target group and data collection tool

Tool	Health professionals	Nharrubes	Community leaders	Traditional birth attendants	Total
SSI	16	10	–	–	26
FGD	–	–	43	44	87
Informal conversation	–	3	4	–	7

FGD = focus group discussion; SSI = semi-structured interview.

Socio-demographic characteristics of the 26 SSI participants are summarized in Table 2. The median age was 53 years (interquartile range [IQR]: 35–62), 62% were female, and 23% of respondents had no formal education. Among the respondents, 35% were farmers, and the vast majority of nharrubes were also traditional healers.

**Table 2:** Socio-demographic characteristics of SSI participants

Characteristics	Male	Female	Total (*N* = 26)
Median age, years (IQR)	54 (37–59)	52 (35–63)	53 (35–62)
Sex, *n* (%)	10 (38)	16 (62)	26
Education level, *n* (%)			
No formal education	0	6 (23)	6
Primary education	5 (19)	2 (8)	7
Secondary/higher	1 (4)	0	1
College (complete or in part)	4 (15)	8 (31)	12
Marital status, *n* (%)			
Never married	3 (12)	5 (19)	8
Married	5 (19)	6 (23)	11
Widow	2 (8)	5 (19.3)	7
Religion, *n* (%)			
Catholic Christian	7 (26)	8 (31)	15
Muslim	2 (8)	2 (8)	4
Protestant Christian	1 (4)	6 (23)	7
Occupation, *n* (%)			
Nharrube			
Farmer	1 (4)	8 (31)	9
Carpenter	1 (4)	0	1
Healthcare providers			
Support staff at health facility	2 (8)	0	2
Social worker	1 (4)	2 (8)	3
Medicine technician	2 (8)	4 (15)	6
Medical doctor	3 (12)	2 (8)	5

IQR = interquartile range; SSI = semi-structured interview.

Table 3 describes the characteristics of FGD participants. The median age was 48 years (IQR: 34–57). Most FGD participants were female (74%), 51% of them were TBAs, 18% did not have a formal education, and 32% had primary education level. Only 2% of the participants had attended higher education.

**Table 3:** Socio-demographic characteristics of FGD participants

Characteristics	Male	Female	Total
Sex, *n* (%)	23 (26)	64 (74)	87 (100)
Median age, years (IQR)	42 (31–53)	49 (35–60)	48 (34–57)
Community role, *n* (%)			
Community leader	23 (26)	20 (23)	43
Traditional birth attendant	0	44 (51)	44
Education level, *n* (%)			
No formal education	0	16 (18)	16
Primary education	8 (9)	28 (32)	36
Secondary	15 (17)	18 (21)	33
College	1 (1)	1 (1)	2
Marital status, *n *(%)			
Never married	14 (16)	25 (29)	39
Married	7 (8)	18 (21)	25
Cohabitating	3 (3)	5 (6)	8
Divorced/widowed	0	15 (17)	15
Religion, *n* (%)			
Catholic Christian	11 (13)	33 (38)	44
Muslim	8 (9)	13 (15)	21
Protestant Christian	5 (6)	17 (19)	21
Occupation, *n* (%)			
Farmer	11 (13)	50 (57)	61
Carpenter	5 (6)	0	5
Dressmaker	1 (1)	3 (3)	4
Driver	2 (2)	0	2
Pastor	2 (2)	0	2
Civil construction assistant	8 (9)	0	8
Retired teacher	3 (3)	2 (2)	5

FGD = focus group discussion; IQR = interquartile range.

### Experiences related to the death of a child.

In general, child mortality was characterized as a sad, striking, and unexpected event. Numerous participants from both SSIs and FGDs mentioned that child deaths are treated in a special way and that they are accompanied by specific itineraries and ceremonies, especially when it comes to stillbirths.

When inquired about specific issues related to stillbirths, all participants emphasized that a stillborn child is not considered to have existed as a human being. Participants recounted that stillborn children are perceived to be children who were “returned to God.” Therefore, some nharrubes have explained that the ceremonies for a stillborn child cannot be performed following the same rituals related to the death of an adult because the stillborn child is not considered a human being who has lived. For example, one man stated that, “a child who was born dead, did not have a life like us, so it cannot be buried normally as we do with an adult person. We don’t need to use a box, we just bury it” (nharrube, male, Namuinho, SSI).

Some participants reported that local norms dictate that the body of a stillborn child cannot be taken to the family home so as to not infect the mother with the evil spirits of death. Thus, small and restricted ceremonies, different from the regular ceremonies of a deceased adult, are held in the home of family members other than the parents of the stillborn.

*When a child is born dead [they] cannot enter in the family home, this is because, you cannot join a dead child with their mother without undergoing a purification treatment so that she [the mother] overcomes the loss, gets pregnant and in the future gives birth to another healthy baby.* (traditional birth attendant, female, field note)

The above quote illustrates the essence of the link between the meaning of a stillbirth and the perceived consequences for the mother and her future pregnancies because the requirements to contain such consequences involve the separation between the dead body and the newly purified body of the mother. According to participants, the mother’s purification consists, first of all, of ensuring that she does not see the baby’s body being buried and later, with prayer and bathing, using specific herbs that serve to separate the living from the dead. This ritual serves to ensure that the baby has a blessed return and that the mother is purified so that she can re-conceive and give birth to a healthy child.

Experiences with the death of children were captured from the perspective of events occurring both within and outside health facilities. After the death of a child, regardless of the place of death, it is common for some community members and leaders to get involved. Participants describe it as an event that is easily disseminated within the community, and neighbors mobilize in solidarity to support funeral ceremonies, which include contributing to the expenses of transporting the body, feeding the family and other participants during funeral ceremonies, and buying a coffin.

*When a child dies everyone gets that sad feeling, then everyone goes to the neighbor’s house to provide solidarity and emotional support.* (community leader, male, FGD)

Even if the death is at the health facility, the child’s parents or caregivers are quickly notified, who in turn pass on the information to one of the community authorities, such as the neighborhood secretary, religious leaders, nharrubes, or TBAs, to lead or support the family in caring for the deceased body (depending on the norms of each family) before the funeral ceremonies start.

Participants reported that some stillborn infants, particularly those belonging to families experiencing financial constraints, are left under the responsibility of the health facility, which in turn mobilizes resources to help dispose of the body in a common ditch because parents are not able to transfer the body back to their community. In these cases, the health facility requests the consent of either parent to conduct the disposal along with the other stillbirth cases.

Between 1 and 3 days after the death of a person, whether child or adult, the funeral is held. The timing varies depending on the religion of the family of the deceased. Participants mentioned that it is common among Christian families to perform the funeral 2 or 3 days after death, whereas in Muslim families the funeral ceremonies must ideally take place before the end of the first 24 hours. For stillborn infants, regardless of religion, the body is buried within the first 24 hours.

Also regardless of religion, in cases of death the presence of the nharrube is crucial. Some participants mentioned that in some families the ceremonies are guided by the nharrube and in others by a religious leader. In many cases a religious leader can also be a nharrube.

In preparation for the funeral, several rituals take place: washing, purification, and blessing. For the washing, the nharrube is authorized to choose a “brave” family member to assist in the process. Purification and blessing take place after washing the body and are performed by a religious leader before burial. After washing, purification, and blessing, the body can no longer be touched by “strangers” (i.e., those who are not direct relatives).

Additionally, nharrubes and community leaders frequently reported that after the funeral and purification ceremonies are over, children’s parents seek out healers to help them learn the causes of their child’s death. This action by the deceased child’s relatives to find out the COD is part of the itinerary to be followed during the time of mourning in some families and serves to end the tensions and accusations of witchcraft among the family members.

### Potential motivators for MITS implementation.

The factors analyzed in this study comprise the features already in place at the individual or community level, mostly related to the already mentioned social and cultural norms related to the death of a child that would act as drivers that could influence positively the acceptance of MITS, namely parents’ desire to know the causes of child deaths and the possibility of acquitting the elderly of witchcraft accusations.

#### Willingness to know the causes of child deaths.

When asked about what would make MITS acceptable, the most frequently cited motivation was the desire that participants expressed in knowing the causes of child deaths. This was repeatedly expressed through FGDs with community leaders and TBAs. Study participants reported that when a child dies, family and community members often do not know what the causes were, particularly if it is a sudden death. MITS, which is considered by participants as an innovation, was seen as potentially helpful in providing clear answers about the COD to the parents of the deceased child: “…it is a good [thing] because from these analyses we’ll know what is killing our children, because here many children are dying without knowing what is the disease that killed them” (nharrube, female, SSI). In addition, and as mentioned earlier, TBAs alluded to the parents’ practice of seeking answers as to what caused the child death, through consultation with traditional healers. In their opinion, MITS would address this desire of parents and family members by providing reliable information regarding the cause of the child’s death.

Knowing the causes of a child’s death through MITS has also been cited by TBAs as an initiative that can help the parents of the deceased child treat and prevent diseases that they could have and that have been diagnosed by the MITS procedure.

Healthcare providers were also motivated by the desire to know the real causes of the deaths of hospitalized children. Their particular concern relates to children that are admitted to hospital in a critical state and whom they cannot save or diagnose on time. Thus, they value MITS because they consider that it could help discover the COD in these cases.

*For us it will be a good thing because it will be a complementary service to help us understand the causes of those sudden deaths.* (healthcare provider, male or female, SSI)

#### Intention to acquit the elderly of witchcraft accusations.

Another potential motivation for accepting MITS was the intention to acquit the elderly of witchcraft accusations when a child dies. Participants expressed that child deaths are often attributed to witchcraft and that the elderly are perceived to bewitch children and kill them to increase their longevity. Participants reported that the charges are made to any healthy-looking elderly individual, especially those with experience as healers.

*With the results of these analyses at least they will stop saying that we the elderly have bewitched the children to die.* (community leader, male, FGD)

Paradoxically to the accusations of witchcraft resulting in children’s deaths, the elderly are recognized to play a significant role in the preservation children’s lives and well-being. Established nharrubes, healers, and TBAs, who are mostly elderly people, do care for sick children as part of their role in the community in addition to caring for the bodies of the deceased children. Some elders mentioned that, with the implementation of MITS, the diagnosis of COD for children will be clear and their role as knowledgeable elders in the community will be credible.

### Expected programmatic influencing factors.

When asked about which factors would facilitate the acceptance of MITS, participants alluded to some requisites that would have to be consigned to the intervention, namely: the facilitation of transportation of bodies to the community, the dissemination of the intervention through the community radio and health talks, and the involvement of leaders in dissemination activities.

#### Provision of means to transport bodies.

Healthcare providers stated that the availability of transport could contribute to the acceptance of MITS. Because many families are unable to transport the body of a dead child from the hospital to their home, as evidenced by the earlier statements referring to parents not claiming the bodies of their children if they die at the health facilities, healthcare providers believe that providing means to transport bodies back to the communities would establish confidence and motivation to accept MITS if a child’s death occurs.

*Of the patients we have received here, many are unable to rent a car to carry the coffin, so if the program could inform that it will help with transport if necessary it would be good, the population may have a motivation to accept it.* (healthcare provider, female, SSI)

Healthcare providers mentioned that providing transportation to transfer the bodies of deceased children from the health facility to their homes had acted as an additional motivation for potential participants to accept MITS. This act can be seen as support for some families in need.

#### Dissemination of information through trusted channels.

According to the participants, the dissemination of information through trusted channels, such as community associations or community health commutes, and adoption of appropriate community awareness activities will boost the acceptance of MITS by the community members.

*For the community to accept, they must know that it is MITS and that it is being implemented. Therefore, you must make a lot of publicity in several campaigns and health lectures.* (community leader, male, FGD)

Additionally, community leaders mentioned that the information and campaigns disseminated by the community radio stations are commonly trusted and adhered to because most members of the community listen to the radio. Thus, if the MITS are broadcasted on local radio, community members will learn about the initiative, understand the purpose of the program, and easily accept that it is carried out on their children.

*If you use the radios you will gain a lot. This city is big, if you go from house to house, you will not be able to finish it, the best thing is to use the radio for everyone to find out about MITS.* (community leader, male, FGD)

#### Involvement of leaders in the dissemination of project information.

Most participants believe that engaging all influential community leaders in the dissemination process can facilitate MITS acceptance. The influence that leaders have over members of the community allows their messages and recommendations to be easily followed and adhered to. Quelimane is under two overlapping powers; thus, many areas are influenced by central government–appointed leaders and, at the same time, by local government–compried of elected leaders who govern the district and municipal territories simultaneously. For this reason, participants mentioned the need to involve all influential community leaders in the dissemination of information related to the implementation of MITS regardless of their political orientation.

*You must involve the leaders, these secretaries, and the regulators [community political leaders] to help as activists in the community.* (community leader, male, FGD)

A community leader explained that MITS will only be accepted if people feel safe while taking part in the intervention and that, according to him, this sense of safety can only be successfully conveyed by community leaders. Furthermore, there is a belief that if something goes wrong in communicating the message or misunderstandings arise, community leaders can help solve and clarify the problem in the community, thereby guaranteeing the re-establishment and maintenance of the desired sense of safety that community members feel when their community leaders are involved in the process.

*The people here will not accept any new project if they do not feel safe, for that, we are the persons who guarantee safety in them because we are influential here in the community.* (community leader, male, FGD)

Moreover, community leaders’ expression of reluctance regarding the implementation of MITS without their active involvement in dissemination the activities is grounded in the belief that if they are not involved, other people will be called upon to fulfill this role in exchange for financial compensation. One community leader (male, FGD) explained: “Our population here will accept the message if it is disseminated by a healthcare provider or a community leader, but if it is an outside person who will make money we will not accept it because we as representatives of the community can divulge that information.”

As described below, it is notable that the leaders’ requirements are related to possible benefits that they can have in return for giving support to the project in the dissemination of MITS in the community but also to a sense of ownership of an intervention pertaining their own community.

#### Incentives for local leaders.

Some of the above notions of leaders’ involvement were expressed by community leaders as a responsibility they held on making decisions on behalf of the community, to the extent of paralleling it to a job: “If we work we can make the parents of children in the community trust and accept MITS” (community leader, male, FGD). For them, the authority that has been entrusted in them must be reinforced at a time when communities need a representative to help them understand and trust the new interventions.

Some leaders were more specific, stating that their involvement and support for COMSA intervention should be rewarded with cash compensation or incentivized with the provision of support material, such as bicycles (for mobility), t-shirts (for identification and credibility in their genuine link to the intervention), and airtime for their mobile phones (to communicate with the study team and the members of the wider community). Other leaders suggested direct collaboration through the hiring of community members to work as COMSA activists in the community, implying some extra income for them.

### Potential barriers to MITS implementation.

The main factors that may constitute barriers to the implementation of MITS were the possible disagreements with Islamic religious practices, skepticism regarding the objectives of the intervention, and negative past experiences with health interventions.

#### Disagreement with Islamic religious practices.

The norms surrounding post-mortem Islamic rituals constitute important factors influencing the potential refusal to perform MITS. Therefore, in the case of the death of a person belonging to the Islamic faith, the aforementioned washing, purifying, and praying rituals must be performed within 24 hours of the death. This is to allow the spirit to be blessed and to reach heaven while clean. Furthermore, the body cannot be dissected or sutured, especially if Islamic religious leaders have already blessed the body. Additionally, if the death occurs in the hospital, it is not acceptable for the body to be exposed for long hours to the hospital environment so as to minimize the spirit’s suffering.

Further, according to participants, if a person belonging to the Islamic community dies, including stillbirths, it is only acceptable for the body to be manipulated by a person who is a family member and a Muslim, unless the person is a nharrube. Nharrubes, despite not being a family member and not necessarily being a Muslim, are highly respected and considered custodians of the rules around body manipulation in Quelimane.

Regarding the implications for MITS implementation, many participants emphasized that timing restrictions marking funeral rites could compromise the achievement of MITS. One Islamic leader explained that:

*When we receive information that an adult person or a child has died, we are the first to arrive [in the family home] to make our prayers. Prayers help the spirit be in peace.* (community leader, male, FGD)

Participants noted that in some cases the achievement of MITS will be compromised because it has the potential to interrupt or delay the washing and grooming of the body by the nharrube, which are considered vital post-mortem practices. As one participant explained: “We may even want this MITS, but we have to follow up with our [Islamic] religious norms to guarantee peace for the spirit” (community leader, male, FGD).

Respondents who expressed difficulties in accepting MITS were mainly Muslims, but some were Christians belonging to non-Catholic churches, such as the Jehovah’s Witnesses. Most participants had high levels of education, such as retired teachers, whom are considered influential people within communities.

#### Skepticism due to negative past experiences with health interventions.

Community leaders mentioned that people are afraid of organ trafficking allegedly carried out by some entities, such as nongovernment organizations operating in the health field, and that this could lead to skepticism regarding the motivations behind the practice of MITS. These fears are based on rumors about the extraction of organs from deceased bodies at local health facilities. For example, they recalled an episode linked to a cholera outbreak that occurred in 2017, during which the body of a cholera victim who had died at the cholera treatment center was returned to relatives wrapped in a white sheet and with the nostrils and ears plugged with cotton buds, an image community members were not familiar with. This episode gave rise to negative reactions among relatives and community members, which culminated in the destruction of the cholera treatment center in Quelimane.

*It is a problem because they [relatives] may be suspicious that they [health professionals] want to take organs as other institutions have already been accused of. So people can refuse to perform these MITS.* (community leader, male, FGD)

Some community leaders and nharrubes also claimed to have little confidence in health authorities because they believe that health institutions may intend to use bodies of deceased children for obscure scientific experiments.

*It is normal to suspect because there is not much confidence in some organizations, especially those private ones that come here to make money. Even people do not trust anything.* (community leader, male, FGD)

They pointed out that this suspicion is what triggers people not to not leave the bodies of their deceased relatives for a long time in the hospital’s mortuary.

Community leaders reported that some health interventions and programs previously implemented in these communities have failed because of low levels of involvement and engagement of leaders in the dissemination of information about the intervention. According to participants, MITS can be easily rejected if the community leaders are not made responsible for conveying information about MITS to the population.

Some study participants mentioned that, due to a history of weak involvement of community leaders in the dissemination of new health interventions in Quelimane, some campaigns have been characterized as inappropriate by the population, an example being the promotion of cervical cancer screening for women of reproductive age, which was erroneously interpreted as a campaign to promote birth control.

Another community leader’s statement illustrates the point that their involvement and support to the dissemination of a future MITS intervention stands out almost as a requirement:

*If you do not call on the leaders of the community here to work with you then this program will not move forward.* (community leader, male, FGD)

## DISCUSSION

The implementation of any postmortem procedure to determine the COD in a social setting that is not familiar with research studies, with some of the poorest health indicators and a history of myths, rumors, and negative perceptions surrounding specific public health initiatives, is marred with challenges.^18^^,^^19^ It is thus evident that the success of the intervention would require an in-depth understanding of what is culturally and religiously acceptable and feasible.^11^^,^^16^ Similar to what has been reported in other settings, prior to the implementation of the MITS procedure, an assessment of the foreseen acceptability of child mortality surveillance was conducted to understand attitudes and perceptions surrounding death as well as possible barriers and facilitators to the implementation of the post-mortem procedure.^6^^,^^11^^,^^20^ This study provides a useful body of evidence on community issues potentially conditioning the implementation of MITS in Quelimane.

Our results suggest that, no different from other settings, the death of an individual is characterized as a sensitive social event.^21^^,^^22^ The death of a child is marked by age-specific rituals that are rooted in local beliefs and is an event that has consequences for the parents (particularly the mother), the family, and the community. These consequences are tied to the specific rituals and itineraries that must be followed by families of the deceased children. According to the findings of this study, stillbirths are conceptualized as children that have returned to God before being fully established and therefore before having reached the status of human beings in their full capacity, a phenomenon that is associated with spirits of death. Thus, there is a certain degree of exceptionalism in how stillbirths are conceived of and socially handled. Specifically, they are handled in accordance with distinct rites and without contact with the closest relatives to guard them from further misfortune. These findings are not unique to Mozambique. Similar results were reported in a study conducted in Ethiopia describing stillbirths and newborns who die soon after birth as non-human. In this case they are also hidden from the community, and the mother of the baby assumes that the baby never existed.^23^

The religious requirements pertaining to a section of this community add to the complexities regarding the death event. The sensitivity of this event is also deeply related to the events that unfold at the community level, such as ceremonies and practices around death. Our data also revealed that the economic constraints that mark the reality of funeral ceremonies in Quelimane play a crucial role in defining bereavement. Community members and leaders often mobilize resources within their social network to support funeral ceremonies and contribute to expenses. Yet, in many cases, the younger the child is, the fewer the resources that are collected, leading families of very young children not to claim the body at the health facilities. Healthcare providers have also reported this situation, stating that it will be more feasible to carry out MITS on stillbirths because these bodies are more likely to be left at the hospital. This situation may increase the likelihood of post-mortem investigation on stillbirths compared with the other age groups, a finding that may be relevant for Mozambique and other countries with similar practices.^24^ Although this can be considered a potential facilitator for the performance of MITS, it must be received with caution because it raises ethical issues. Also, community awareness of the practice of MITS on “abandoned” bodies could potentially escalate into rumors of hidden research agendas similar to previous experiences related to cholera in the same context, where the body of a cholera victim who had died at the cholera treatment center was released while wrapped in a white sheet and having the nostrils and ears covered with cotton buttons; the relatives believed that the nostrils and ears were covered because the organs had been extracted.

The findings of this study suggest that MITS would generally be accepted by parents who experience the loss of a child. This aligns with results from a qualitative study carried out in five sites in Africa and Asia, which found a high hypothetical acceptability of MITS for COD determination.^11^ However, these results differ from the findings of a previous study that suggested low levels of hypothetical acceptability of post-mortem procedures to determine the COD, particularly in Muslim communities.^21^

The results from our study revealed different factors that constitute potential motivators for acceptance of MITS, namely 1) parents’ willingness to know the cause of death to prevent future disease episodes in their children, 2) the desire to gain answers that explain sudden deaths, 3) the need to restore the reputation of elders that are often blamed for child deaths in their communities, and 4) health professionals’ anticipation of MITS as a procedure that can help uncover the true COD of children who had been admitted in a critical condition. In addition, study findings anticipate other factors that, if introduced, could further enhance the acceptability of MITS once implemented. We refer to these as “programmatic factors,” and they include 1) the establishment of acceptable mechanisms of information, communication, and education and 2) logistical arrangements to incentivize leaders and community members alike.

Participants believe that in the event of a child’s death, some relatives are willing to know the COD. In comparison, studies conducted with parents and family members of children who died and a MITS procedure was offered showed that the experienced acceptability was driven by the opportunity to prevent further deaths of children in the family.^9^^,^^10^^,^^12^ Of note, this potential facilitator of anticipated acceptability of MITS in Quelimane is to some extent influenced by certain cultural norms, as the findings of our study revealed that some community members already practice a desire to know the COD following local practices, such as consultation with oracles through healers. This suggests that the introduction of MITS in this context could be in alignment with some of the existing practices surrounding the death of a child. Similar results were found in a study conducted in Tanzania and Chile, where after the death of a child or an adult their relatives seek to know the COD through trusted healers.^2^ However, this possible alignment should be interpreted with caution because MITS intervention can be seen as a competitor to traditional healers’ role to establish the COD.^26^

In the present study, the willingness to know the COD is sometimes tied to the desire to discharge the elderly of accusations of witchcraft related to infant deaths, and therefore community leaders mentioned it as an important factor that could facilitate the acceptance of MITS. In this case, the indictment is influenced by the experience of some elderly persons as former healers and their longevity. A study in Tanzania also showed that the accusation of witchcraft weighs more heavily on the elderly, especially in older women, who are often also healers.^27^ Similarly, it is important to consider that, in cases where the MITS procedure is not able to provide clear results that satisfy the expectations of individuals, the communication of results could have unintended effects. One possible unintended effect could be that the contrary outcome is achieved and that the accusation of witchcraft is reinforced. This possibility was not discussed by participants, yet it has the potential to become a point of tension during future MITS implementation, especially considering the importance given to accusations of witchcraft.

More extensive and meaningful engagement with leaders and other influential people during community mobilization activities may enhance MITS acceptance. However, it is important to note that in Quelimane, where there are community leaders from different political parties, this approach may be particularly necessary for the successful implementation of the MITS. Even so, it is also worth considering that the engagement of community leaders is sensitive to the nuances of their roles as respected actors that wield significant influence and as elders that are susceptible to witchcraft accusations due to the perceived association between child deaths and elders’ (presumed) desire for longevity.

Our study findings also reveal factors that constitute potential barriers to the acceptability of MITS. These can be organized into those that anticipate a clash between cultural norms and the procedures of the intervention (e.g., disagreement with religious practices) and those that anticipate the social consequences of a history of programmatic failures (e.g., poor community mobilization and the foreseen skepticism toward the agenda behind the implementation of the MITS).

Some of these results are similar to those observed in a multicenter study carried out in Mozambique, Gabon, Kenya, Mali, and Pakistan, which provides evidence of local practices and socio-cultural and religious norms that regulate the manipulation of lifeless bodies and how these influence the hypothetical acceptance of MITS in these contexts.^11^ This study also described concerns about delays in the initiation of ceremonies and burials, highlighting potential barriers to accepting MITS. As reflected in our study findings, concerns with the disruption of burial timings are more pressing in the case of Islamic practices where the timeframe between death and burial is narrower. For Islamic persons, the exposure and delay for burial is considered a disrespect to the spirits of the deceased. This result has been suggested in other studies, on hypothetical and experienced acceptability alike, conducted in Muslim countries.^18^^,^^22^^,^^28^^,^^29^ However, the implementation of MITS-based mortality surveillance programs has been shown to be feasible even in predominantly Muslim countries, such as Bangladesh and Mali.^22^

A notable finding of this study is the role that mistrust may play in the anticipated acceptance of the MITS given the backdrop of organ and human blood trafficking rumors that drove the refusal of past healthcare programs in this context. In Zambézia province, and in Quelimane city in particular, the rumors generated in relation to organ and human blood trafficking are some of the toughest challenges faced by local health programs.^27^^,^^30^^,^^31^ This result was also highlighted in a hypothetical acceptability study that referenced potential barriers to MITS acceptance that were related to fear of organ and blood harvesting during the procedure.^32^ Studies conducted in places where MITS was already being implemented also found that mistrust in medics was an important barrier.^9^ This finding calls for important investments in activities, such as rumor surveillance, which help to detect and trace the circulating rumors in the community an confront them with trustworthy information targeted at the appropriate audiences.^19^

The level of involvement of the child’s parents, religious leaders, and nharrubes may be key to the success of the acceptability of MITS in Quelimane. Moreover, in line with other studies,^4^^,^^11^ our results show that the involvement of community structures and the consistent desire to know the COD can also influence the acceptability of MITS, even when this desire is fraught with beliefs that include differentiated practices, fear, anxiety, worry, despair, and sadness. Overall, this study contributes with valuable knowledge to the implementation of child mortality surveillance in Quelimane, Mozambique.

### Limitations.

This study has a few important limitations that may have influenced the trustworthiness of the data. The first limitation is that these are hypothetical scenarios in which the participants answered what their reaction would be if they were approached with a request for consent to conduct MITS on their deceased children or how they thought members of their communities would react to the same request. In this sense, results must be interpreted as what is anticipated by participants rather than as what de facto would have happened. However, an important number of anticipated barriers and facilitators to MITS found in this study coincide with those elucidated by studies that were conducted in real scenarios of MITS implementation, adding assurance of the relevance of our results to inform future MITS implementation in Quelimane.

In this study, data collection was conducted by a team of CISM research assistants operating under Ministry of Health credentials. In addition, CISM and the Ministry of Health are seen as highly regarded entities that must be respected, which also may have influenced participants’ choice of information conveyed to the researchers. Their willingness to assist the Ministry of Health and CISM in the implementation of MITS, as well as their role as gatekeepers together with the study’s snowballing sampling strategy, may have had an impact on the overall profile of study participants. Therefore, there were gains as well as limitations in obtaining data from privileged participants. Lastly, study participants were people with power and influence in the community or who held some publicly important roles in the community; therefore, the opinions of the “ordinary” members of the community were lacking, calling for further studies targeting this group.

## CONCLUSION

The results of this study highlight important factors associated with the potential acceptance and refusal of MITS in Quelimane. Although some of the respondents considered post-mortem procedures complex because of religious and traditional norms, participants also expressed a desire to accept MITS to know the children’s COD. Although MITS was considered a positive innovation to determine the COD in children, participants remain skeptical about the procedure in lifeless bodies due to tensions with religion and tradition that include the fear of delays in funeral practices particularly regarding Muslim ceremonies. However, although the identified negative and positive aspects have the potential to influence the acceptability of MITS, the participants’ desire to know the COD of children was perceived as the most recurrent factor associated with the acceptability of MITS.

Thus, the implementation of MITS in Quelimane should prioritize the involvement of a variety of influential community and religious leaders, as well as transparency in terms of the information provided to family members, in addition to the need for a truthful dialogue with the direct relatives of deceased children. The findings of this study could thus prove useful in the optimization of childhood mortality surveillance using MITS to determine COD in Quelimane.

## Financial Disclosure

Financial support: This study was funded by the Bill & Melinda Gates Foundation under the Grant OPP1126780 to Robert Breiman, subcontract SC00003286-S1, via CHAMPS Network. CISM is supported by the Government of Mozambique and the Spanish Agency for International Development Cooperation (AECID).
